# Dogs can discriminate between human baseline and psychological stress condition odours

**DOI:** 10.1371/journal.pone.0274143

**Published:** 2022-09-28

**Authors:** Clara Wilson, Kerry Campbell, Zachary Petzel, Catherine Reeve

**Affiliations:** 1 Animal Behaviour Centre, School of Psychology, David Keir Building, Queen’s University Belfast, Belfast, United Kingdom; 2 School of Psychology, Newcastle University, Newcastle upon Tyne, United Kingdom; Helsingin Yliopisto, FINLAND

## Abstract

Previous research suggests that dogs can detect when humans are experiencing stress. This study tested whether baseline and stress odours were distinguishable to dogs, using a double-blind, two-phase, three-alternative forced-choice procedure. Combined breath and sweat samples were obtained from participants at baseline, and after a stress-inducing (mental arithmetic) task. Participants’ stress was validated with self-report and physiological measures recorded via a Biopac MP150 system. Thirty-six participants’ samples were presented to four dogs across 36 sessions (16, 11, 7 and 2 sessions, respectively). Each session consisted of 10 Phase One training trials and 20 Phase Two discrimination trials. In Phase One, the dog was presented with a participant’s stress sample (taken immediately post-task) alongside two blanks (the sample materials without breath or sweat), and was required to identify the stress sample with an alert behaviour. In Phase Two, the dog was presented with the stress sample, the same participant’s baseline sample (taken pre-task), and a blank. Which sample (blank, baseline, or stress) the dog performed their alert behaviour on was measured. If dogs can correctly alert on the stress sample in Phase Two (when the baseline sample was present), it suggests that baseline and stress odours are distinguishable. Performance ranged from 90.00% to 96.88% accuracy with a combined accuracy of 93.75% (N trials = 720). A binomial test (where probability of success on a single trial was 0.33, and alpha was 0.05) showed that the proportion of correct trials was greater than that expected by chance (*p* < 0.001). Results indicate that the physiological processes associated with an acute psychological stress response produce changes in the volatile organic compounds emanating from breath and/or sweat that are detectable to dogs. These results add to our understanding of human-dog relationships and could have applications to Emotional Support and Post Traumatic Stress Disorder (PTSD) service dogs.

## 1 Introduction

Odours emitted by the body constitute chemical signals (chemosignals) that have evolved for communication, primarily within species [1]. A canine’s sense of smell provides critical information, essential for being aware of potential predators, locating food, identifying conspecifics (and their reproductive status), and enabling recognition of familial members [2–4]. Research on chemosignals has extended to explore inter-specific communication, such as that between mice and humans [5], cows and humans [5], horses and humans [6] and canines and humans [7]. Given domestic canines’ remarkable sense of smell, and their close domestication history with humans, it is possible that they are detecting odours associated with changes within the human body beyond those that have already been established. The use of dogs to support human psychological conditions such as anxiety, panic attacks and Post Traumatic Stress Disorder (PTSD) is growing in popularity, with waiting lists for PTSD service dogs being months-to-years long in some instances [8]. Such dogs have been reported to improve an individual’s quality of life, social connections, and reduce the number of panic attacks or PTSD symptoms [9, 10], with the tasks of ‘calming’ and ‘interrupting anxiety’ reported as the most helpful part of their behavioural repertoire [11]. However, empirical evidence for what mechanisms dogs may be utilising to respond to their owner’s psychological experience is currently lacking.

The principal physiological process associated with anxiety, panic attacks, and PTSD is the stress response. In humans, stress is associated with several physiological changes, including epinephrine and cortisol release into the bloodstream, in addition to increased heart rate, blood pressure, and respiration, with suppression of digestion [12, 13]. While there are many definitions of stress, it is broadly defined as a physiological and psychological response to a challenging situation (i.e., a stressor) which is exacerbated when an individual does not feel confident in their ability to overcome the stressor [13]. The biopsychosocial model of stress [14–16] provides a theoretical framework for a connection between psychological states and physiological responses. This framework is underpinned by the premise that psychological processes lead to physiological changes, and that these physiological responses occur in a matter of seconds, affecting the functioning of the cardiovascular system [16]. Using the biopsychosocial model of stress, an individual’s response to stress can be categorised as either negative (threat) or positive (challenge) [14]. Negative responses to stress occur when an individual does not believe they have the resources to overcome a stressor and is accompanied by increased heart rate and blood pressure, whereas a positive stress response occurs when an individual believes that they do have adequate resources or ability to overcome a stressor and is often accompanied by a decrease in blood pressure [15]. While stress is not conceptualised as an emotion, in humans, negative stress (threat) does elicit relatively automatic emotional responses based on an individual’s perception of a stressor, which engages brain regions implicated in experiencing fear [17] and can produce some of the same physiological responses as fear [18].

Previous research looking at non-human animals’ perception of human psychological states have primarily focussed on fear and happiness, or valences such as ‘positive’ or ‘negative’ when assessing intra- and inter-specific communication, largely through visual and auditory cues. For example, horses [19] and goats [20] were able to discriminate between images of human faces with different emotional expressions. Dogs have been reported to recognise conspecific and human emotions based on both acoustic and visual cues by using a cross-modal preferential looking paradigm [21]. Recently, research has extended to transmission of emotional information via odour signals between species, such as a recent study by Destrez et al. [5] finding that male mice and cows seemed to perceive and react to stressful human chemosignals. As olfaction is key to a dog’s perception of their environment and those around them [22], it is imperative to investigate human-dog relationships through the lens of olfaction. Given that dogs possess an acute sense of smell [23], and have been demonstrated to be able to detect changes in human physiology through odour (e.g., seizure odour [24]), it is feasible that olfactory cues may also be associated with human emotional and psychological states, for example, acute negative stress.

Dogs’ detection of human psychological states has, thus far, primarily been assessed via emotional contagion. Emotional contagion describes a process whereby the emotional or arousal states between individuals is mirrored. This phenomenon is seen among group-living species, predominantly within species [25]. However, recent evidence suggests that emotional contagion occurs across species living closely to one another, such as dogs and humans [26, 27]. Sundman et al. [28] report that the long-term cortisol level of pet dogs’ mirrors that of their owners. This finding was unrelated to exertion or exercise, suggesting that the cortisol levels were a product of psychological, rather than physical, stress. However, the mechanisms by which dogs may be detecting their owner’s stress were not examined. It is possible that a combination of verbal, visual and olfactory cues may communicate the owner’s level of stress to their dog. D’Aniello et al. [7] sought to see if dogs would respond differently to sweat taken from unknown individuals while experiencing either ‘positive’ or ‘negative’ emotional states, induced by participants watching either ‘happy’ or ‘scary’ videos, corroborated by self-report measures. Here, the sweat of a person experiencing either “happiness”, “fear”, or “neutral” emotions were presented to dogs in a room with both their owner and a stranger (not the person who provided the sample) present. It was reported that dogs showed more stress-indicative behaviours and were less likely to approach the stranger in the condition where the “fear” sweat was presented. D’Aniello et al.’s [7] results suggest that dogs can detect human psychological states, in this case, primarily from olfactory cues, as evidenced by their untrained behavioural responses. However, this study did not record any physiological measures from the human participants during sample collection, therefore confirmation that the sweat samples collected in the “happy”, “fearful”, and “neutral” conditions represented physiologically distinct states associated with each condition could not be established. Further, by introducing the owner and a stranger into the room there were additional extraneous odours that may have contributed to the dogs’ responses. In sum, Sundman et al. [28] and D’Aniello et al. [7] provide evidence to suggest that dogs are able to detect odours associated with human stress, however, what is lacking is a controlled olfactory study that answers the fundamental question of whether dogs can discriminate between human odour samples taken when neutral and under stress.

Dogs’ capacity to communicate via trained behaviours allows an alternative method of investigation to be explored: the bio-detection paradigm. This paradigm allows researchers to address, in a controlled setting, whether odours are distinguishable from one another through the interpretation of a trained dogs’ responses to samples. Reports of dogs’ abilities to detect human conditions have historically emerged first as anecdotes, which have later been corroborated by in-vitro laboratory studies (e.g., diabetic glucose fluctuation: [29–31] and epileptic seizure: [24, 32]). When considering dogs’ abilities to detect stress in humans, it has been reported in the media that dogs can sense when the humans around them are experiencing stress (e.g., [33, 34]), and a recent study by Reeve et al. [35] found that, in a questionnaire to owners of trained Medical Alert Dogs, stress was the most common condition to which dogs were reported to alert. The current study addresses this reported phenomenon using a controlled, in-vitro, bio-detection paradigm.

The canine bio-detection paradigm has been used previously to assess various human health conditions, including epileptic seizures [24], hypoglycaemia [30], bladder cancer [36], colorectal cancer [37], lung cancer [38, 39], ovarian cancer [40], prostate cancer [41, 42], Clostridium difficile (C. diff) [43], and, recently, SARS-CoV-2 infection (COVID-19) [44]. To do this, a ‘scent-wheel’ or ‘line-up’ is used, where the dog is exposed to several human biological samples and trained, through positive reinforcement, to indicate the target odour by performing an alert behaviour on the sample that is indicative of that health condition. The dog is then tasked with discriminating between target samples and other samples taken from controls, such as healthy individuals or individuals who have a similar health condition. It should be noted that this paradigm does not allow insight into a dog’s untrained response to an odour; however, it does offer a level of control that is desirable when attempting to address the question of whether a physiological process confers a detectable odour. Most large-scale human disease studies use a ‘generalisation paradigm’ whereby dogs are taught their target odour by being shown many samples taken from people who have the same health condition (e.g., [45]). Over repeated exposure, the aim is that dogs learn to recognise commonality associated with the condition across the samples and can ignore individual differences unrelated to the condition (e.g., age, sex, diet, medications). Outside of the human disease literature, an array of paradigms have been utilised to assess aspects of canine odour perception, such as odour threshold tests [46], memory for odours [47], ability to discriminate between odours [48] and ability to ‘match-to-sample’ [49]. Thus far, these paradigms have been most commonly been used in conjunction with non-human odours (e.g., amyl-acetate, isoamyl-acetate). However, an interesting area for emerging studies is applying these paradigms, previously used primarily with non-human odours, to a human biological odour setting. Integrating these types of paradigms into the field of dogs detecting human chemosignals could aid in addressing core questions. This type of fundamental work is necessary to definitively answer whether there is an odour associated with psychological stress in humans that dogs are able to detect and, further, that these cues do not need to be in the context of visual or auditory cues to be detectable.

The canine bio-detection paradigm is underpinned by the premise that human body cells release volatile organic compounds (VOCs) that are exhaled in the breath, emanated from the skin, found in urine, and faeces and saliva [50]. Different VOC profiles can be signatures of physiological changes [51]. When considering the VOC profile of stress in humans, breath and sweat have been the sample substances most focussed upon. de Groot et al. [52] found that the sweat collected from participants when they were experiencing stress contained more volatiles than sweat collected during neutral conditions. Turner et al. [53] hypothesised that people experiencing acute psychological stress breathe faster, their pulse rate increases and their blood pressure elevates, which likely confers a change in their VOC profile. Their study found six VOCs of importance associated with stress (induced by the Paced Auditory Serial Test, a cognitive test designed to induce stress) when the participant’s breath samples were processed using Gas Chromatography-Mass Spectrometry (GC-MS). Santos et al. [54] confirmed distinctive VOC changes in exhaled human breath where participants were either in a relaxed or stressed condition. A Paced Auditory Serial Test was, again, used as the stress inducing task and samples were processed using Gas Chromatography-Ion Mobility Spectrometry (GC-IMS). Six VOCs of importance were associated with the breath exhaled in the stress condition, however, these were not the same VOCs identified by Turner et al. [53] (there was some agreement, for example, Benzaldehyde, which appears in both analyses). The issue of different VOC analyses reporting distinctive VOCs of importance is an ongoing challenge in this, and other, areas of human health research (e.g., cancer [55]). While the exact VOC profile of human stress remains inconclusive, the concept of training a dog to assess whether they can categorise a sample as taken during a stress or baseline condition is independent of whether the specific VOCs of importance are known to us or not. If a dog can discriminate between samples taken in a controlled setting, it provides further evidence that there are different VOCs expressed when an individual is at baseline and when experiencing stress. Given dogs’ established ability to discriminate between other physiological processes in the human body, it is conceivable that dogs can detect VOC changes associated with acute psychological stress.

The aim of this study was to determine whether dogs trained on a scent discrimination paradigm could discriminate between the breath and sweat combined samples taken from human participants at baseline and when they were experiencing an experimentally induced state of psychological threat. As outlined previously, threat occurs when the demands of the stressor exceed perceived personal resources and is associated with an increase in heart rate and mean arterial blood pressure [15]. Induced threat can be achieved by using a validated protocol, such as the Mental Arithmetic Task (MAT) [56], which has been extensively used in the human psychology literature. For the present study, sweat and breath were chosen as the sample type to collect pre- and post-task because of previous research indicating that stress inducing tasks produce a measurable change in VOC profile found using these excretions [52–54, 57]. Because stress is not directly or reliably measurable, investigators rely on physiological markers (heart rate, blood pressure, hormones) together with self-report questionnaires to quantify stress [58]. Physiological markers are important to include as they do not require cognitive processing and are more objective than self-report measures [59]. In the current study, to ensure samples were collected from those experiencing a negative response to stress (i.e., threat), the samples were validated using cardiovascular measures (e.g., heart rate and blood pressure), which is a novel contribution to the existing literature surrounding dogs’ detection of stress. If dogs trained on an olfactory discrimination task can successfully distinguish between baseline and stress odour samples, it provides evidence that there is a VOC change associated with the physiological stress response that produces an odour detectable by dogs.

## 2 Methods

### 2.1 Ethical statement

The study protocol was approved in December 2019 by the QUB Research Committee (EPS 19_256). Dog training and data collection were paused from March 2020 to January 2021 due to COVID-19 restrictions. A research protocol amendment to conduct the stress inducing protocol online, and collect samples remotely, was approved in March 2021 so that data collection could go ahead while adhering to UK COVID-19 guidelines. By August 2021, restrictions had eased to the extent that the original human sample collection protocol (conducting the protocol in-person with physiological measures) could take place. Personal Protective Equipment (PPE) were added and the protocol was additionally approved by the University Safety Services for Campus Risk Assessment Guidance. Written consent was obtained by all participants and dog owners.

### 2.2 Human samples

#### 2.2.1 Training samples

During training stages one and two, samples were collected from the researchers and others known to the researchers within three hours prior to the dog arriving for training. Approximately 20 individual’s samples were used during training. No stress-induction protocol was used as the dogs were learning the two-phase, quasi match-to-sample, paradigm (see section 2.5: Training).

#### 2.2.2 Testing samples: Recruitment

Participants providing test samples were recruited via social media (Facebook, Twitter, Instagram), a university-wide email, Sona Systems^®^ (a departmental system for undergraduate students to sign up for studies to receive course credit), and word-of-mouth. Participants were either given £10 cash or Sona credits (if they were a first-year Psychology student at QUB) for taking part. While following the COVID-19 restrictions and guidance at the time, 13 participants completed the study using a remote protocol and 40 participants completed the study using the in-person protocol.

#### 2.2.3 Exclusion criteria

Because of the impact of tobacco, e-cigarette and food consumption on VOC profiles in exhaled breath [60, 61], participants had to be non-smokers and were instructed to withhold from eating or drinking flavoured beverages for a one hour prior to taking part in the study. A further inclusion criterion was that the participant was not on mood altering medication, or, if they were, that they could safely refrain from taking the medication for a minimum of one hour prior to taking part in the study.

### 2.3 Protocol

#### 2.3.1 Remote protocol

Due to COVID-19 government guidelines in place at the time of data collection (24/06/21-12/08/21), 13 participants’ data were collected remotely so that social distancing could be maintained. Because participants were not permitted into campus buildings, sample kits were delivered to the participant’s homes and the experimenters conducted the stress induction protocol over online meeting software (Microsoft Teams or Zoom). Each sample kit was sealed in an air-tight plastic container and was comprised of six vials (marked as: B1, B2, B3, D1, T1, T2, where B = blank, D = distractor and T = target), two packets of gauze (Qualicare 7.5x7.5cm four ply non-woven sterile gauze swabs), and a pair of nitrile, powder free, gloves (Unicare non-sterile, single use examination gloves).

The study was advertised as a mild-stress inducing task, however, the specifics of the task, such as the fact that it involved mental arithmetic, were not made explicit prior to taking part. This was done as an effort to prevent participants’ experiencing stress prior to the task starting, and to minimise participants selecting to take part on the basis of their perceived ability at mental arithmetic. Participants who had expressed an interest in taking part were contacted via email with a meeting link and an online survey link (hosted by Qualtrics). When the meeting began, participants were instructed to click on the link for the survey. The survey first presented participants with the study information sheet and consent form, which they were required to read, and provide written consent, before progressing. Next, participants were required to confirm, via a question in the survey, that they were non-smokers, and had not consumed food or drink (other than water), or any mood-altering medication, for a minimum of one hour before the meeting. Participants then answered demographic questions in the survey including their age, gender, and ethnicity. The virtual meeting was hosted by two researchers. The first researcher asked the participant to put on the nitrile gloves and showed them how to prepare the blank samples, with the first researcher using an example sample kit in front of their camera as a demonstration. Here, participants put on the nitrile gloves, opened a package of sterile gauze, placed a piece of gauze in each of the three vials labelled B1, B2, and B3, and secured the lids. Participants were then shown how to make their baseline sample by wiping a piece of gauze on the back of their neck, placing it in the vial labelled D1, and then exhaling deeply into the vial three times before securing the lid. Participants were then instructed to complete the first self-report measure in the online survey, which assessed their baseline level of stress. The self-report measure was a visual analogue scale (VAS) that presented the statement: “Check along the scale below to indicate the level of stress you are feeling right now on a scale from 0 = none to 10 = the most severe imaginable”. Below the statement was a sliding bar along a line with endpoints 0 and 10, and participants rated their level of stress using the sliding bar. The experimenter then explained to the participant that they would be completing a Mental Arithmetic Task (MAT) where they would be asked to count backwards from 9000 in units of 17, out loud, in front of the two researchers without the aid of a pen or paper. Participants then completed the MAT. As the participant was counting aloud, both researchers used prompts such as “it is very important that you perform the task as quickly and efficiently as possible”, and “you must keep going until the task is completed” [56, pp72] in a stern tone of voice. If the participant gave a correct answer, they were given no feedback and were expected to continue, and if they gave an incorrect answer the researcher would interrupt with “no” and tell them their last correct answer. The task continued for three minutes, regardless of how many correct answers the participant provided. Then, using the same protocol described above for the baseline (distractor) sample, participants were instructed to collect their stress (target) samples. Here, participants collected two samples immediately after one after another, placing them in the vials labelled T1 and T2 (target). Once sample collection was complete, participants were instructed to complete the second self-report measure in the online survey, which assessed their post-task level of stress. All samples were placed back in the plastic container to be collected by the researchers and the gloves were disposed of. Participants were then instructed to read the debrief form in the online survey, and were given a second opportunity to provide written consent to having their data and samples used in the study, now that they had full understanding of what the stress induction task involved. Researchers collected the sample kit from the participant within thirty minutes of the protocol being completed.

Samples were shown to a dog within three hours of being collected. However, samples were used only if they met the following criteria: the participant’s self-report VAS score had to have increased at least two points from baseline to after the MAT. If the participant reported anything less than a two-point increase the samples were disposed of and not shown to a dog, as the task had failed to induce stress in that individual.

#### 2.3.2 In-person protocol

**Fig 1:**
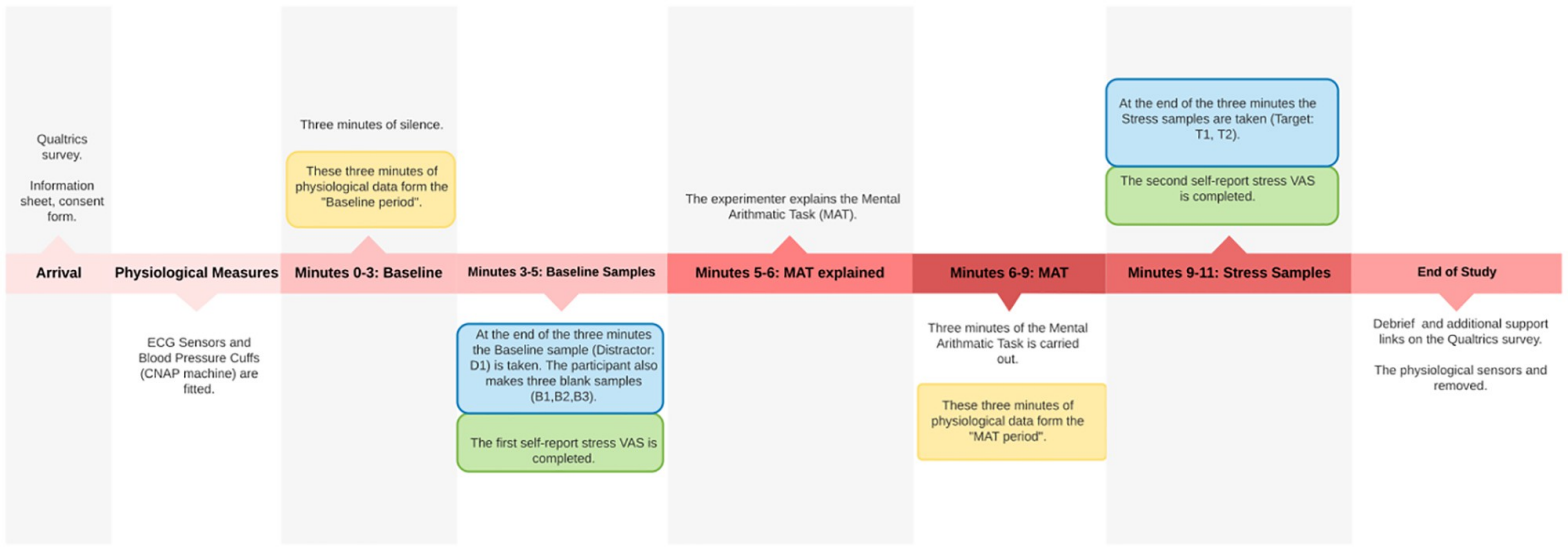
Timeline of the data collection process for the in-person protocol. Yellow highlight shows physiological data used for assessment of stress. Green highlight shows self-report stress data. Blue highlight shows sweat/breath samples that would be later shown to a dog if the participant met the criteria of stress.

During data collection, COVID-19 government advice changed to permit in-person data collection. Therefore, a further 40 participants completed the protocol on campus and in-person, with the addition of physiological measures. Participants completed the survey on a provided laptop in an on-campus study room containing the physiological measuring equipment. Participants were provided with an information sheet and consent form via the same Qualtrics website as in the remote protocol, amended to include content specific to the physiological measures (e.g., “Please pause here. The researcher will now attach the ECG sensors and heart rate monitor”). Physiological data were acquired at 2000Hz through AcqKnowledge software and MP150 system (Biopac Systems, Goleta, CA) using RSPEC and NIBP100D modules. Electrocardiography (ECG) assessed heart rate and was measured with electrodes placed on the right collar bone, bottom of the right rib cage, and bottom of the left rib cage. Mean arterial pressure was used to index blood pressure and was measured using inflatable cuffs placed on the middle and index fingers on the non-dominant hand, with an additional cuff around the non-dominant upper arm to calibrate finger cuffs. Physiological measurements were continually recorded from minute 0 to minute 11 of the study protocol (Fig 1). Once the physiological sensors were in place, a three-minute baseline period commenced, where participants were instructed to sit silently while the machines recorded their baseline measurements. At the end of the three minutes, participants provided the first self-report measure of stress using the self-report VAS and the first set of sweat/breath samples were collected (D1) (in the same manner described in section 2.3.1). From here, the protocol followed that of the remote protocol, with the addition of removing the physiological sensors at the end of the study (for the full protocol timeline see Fig 1). Two time periods were of specific interest during the 11-minute protocol when assessing the physiological data: minutes 0–3 (baseline period) and minutes 6–9 (MAT period).

For these samples to be shown to a dog, the criteria were: a two-point increase in self-report stress from the self-report VAS *and* an increase in the mean heart rate (HR) and mean arterial pressure (blood pressure: BP) when comparing the average of each during the baseline and MAT period. Each participant’s VAS score, mean HR and mean BP were checked for these criteria before showing the samples to the dog. If a participant did not meet all three criteria, their samples were disposed of.

### 2.4 Study dogs

#### 2.4.1 Dog recruitment

Pet dogs from the Belfast community were recruited via posters, an email to QUB staff and students, social media (Twitter and Facebook) and word-of-mouth. Dogs were required to have been vaccinated and to demonstrate basic obedience skills. Dog owners were invited to the laboratory for the first session to allow them to read the information sheet and ask any questions. If they were happy for their dog to be involved in the study, they signed a written consent form and provided necessary details such as dietary requirements and veterinarian contact details in case of an emergency. After this initial session, dog owners were not present in the laboratory during further training and testing sessions to avoid extraneous cues or distractions. Dogs had access to fresh water at all times. A total of 20 dogs engaged in some training and four reached the testing phase. Of the 20 dogs that had training, two dogs were unable to continue with training because their owners were shielding from COVID-19, three dogs were excluded for behaviour reasons (the researchers interpreted stress-indicative behaviours from the dogs when their owners left), and nine dogs started to show behaviours of disinterest (e.g., lying down between trials, slow to approach the apparatus) as training progressed, or were unable to achieve our performance criteria in stage one (between people discrimination) and stage two (within person discrimination) of training (see Sections 2.5.2 and 2.5.3). A further two dogs were progressing through the training stages but had not reached the testing stage by the time that the study ended.

The four dogs that took part in testing ranged in age from 11 to 36 months (Mean = 27 months, 2.25 years) and consisted of a male Cocker Spaniel, a female Cockapoo, and two undetermined Mixed Breeds (one male Lurcher-type and one female Terrier-type). The Cocker Spaniel and Cockapoo were raised by their owners from the age of ten weeks and the Mixed Breed dogs were rehomed from rescue centres. All dogs were sterilised.

### 2.5 Training

#### 2.5.1 Indication behaviour on breath and sweat samples

**Fig 2:**
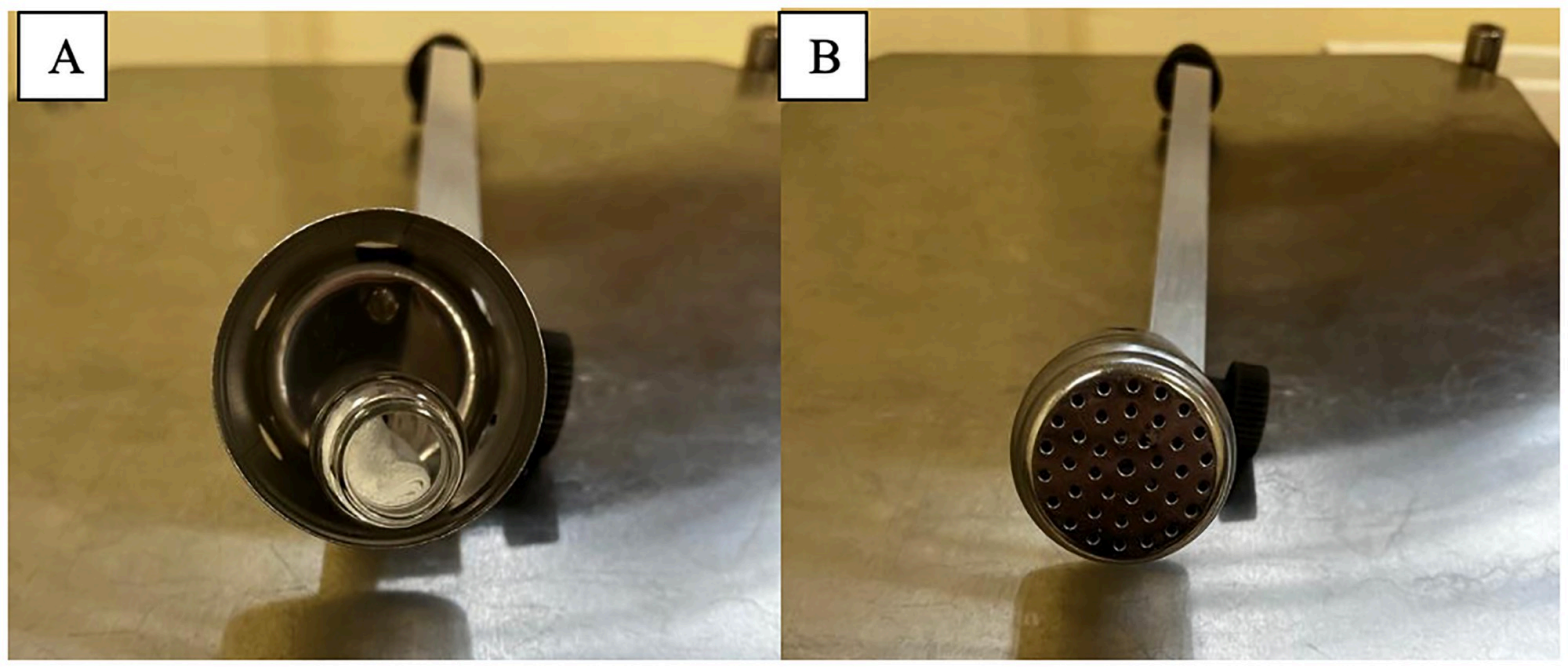
A) A single arm of the apparatus with an open port holding a sample. B) A single arm of the apparatus with the port secured by a lid.

**Fig 3:**
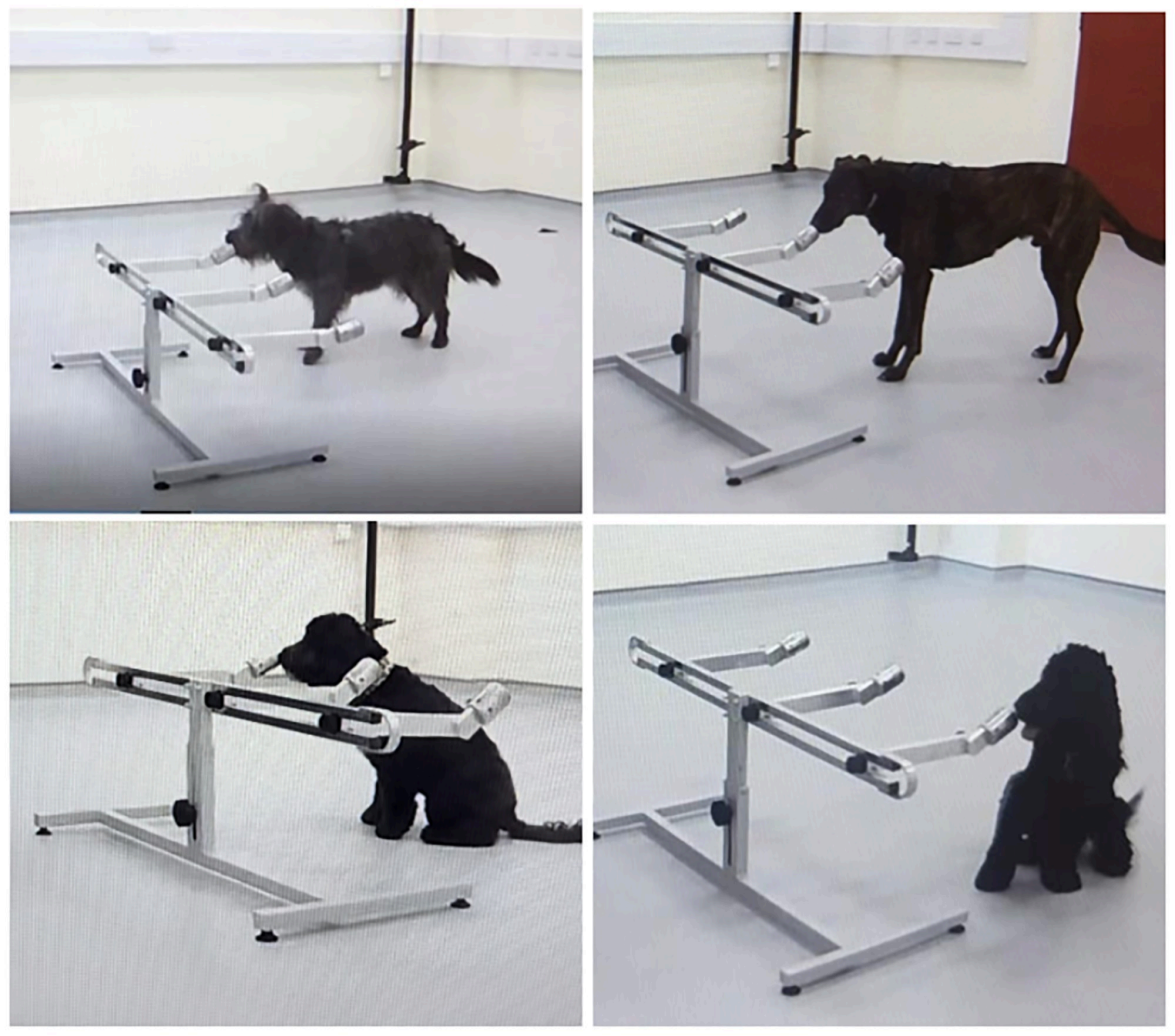
Each dog performing their alert behaviour to indicate their choice on the three alternative forced choice apparatus. Top Left: Soot (stand-stare), Top Right: Fingal (stand-stare), Bottom Left: Winnie (nose-on sit), Bottom Right: Treo (nose-on sit).

The apparatus used for training and testing was purpose-built for the study and consisted of three, removable, aluminium arms that extended from a central frame. The end of each arm had a stainless steel cylindrical ‘port’ with a removable lid (Fig 2). The main body of the apparatus could be adjusted for each dog’s height (see Fig 3).

All dogs were trained using operant conditioning and positive reinforcement. A secondary reinforcer (a clicker) was used to shape the dogs’ indication behaviours and food was used as the primary reinforcer (the dog’s kibble provided by their owner, kibble bought and stored in the lab, cheese and sliced chicken). The dogs were first trained, using shaping and positive reinforcement, to provide an indication behaviour. During this phase of training, practice breath/sweat samples were collected from a member of the research team and placed inside a designated practice port along with a small piece of food. The dogs were trained to provide a nose-hold indication (standing at the target port with their nose close to or touching the port for five seconds). For some dogs, frustration behaviours (e.g., vocalisations, biting the port) were observed when they were presenting their alert using a stand-stare behaviour, so these dogs were trained to perform nose-on sit indication instead (sitting in front of the port with the nose touching the port for five seconds) (Fig 3). In light of Essler et al.’s [62] study suggesting that different types of alert behaviours may impact laboratory scent detection performance, a nose-on sit was chosen rather than a sit away from the port as this allowed the dog’s nose to remain close to the sample, continuing to provide odour information, while making their indication decision. When the indication behaviour was sufficiently developed, the food port (containing a breath and sweat sample with food) was placed on the apparatus with two other ports holding blank samples (glass vials containing unused gauze) and the dogs were allowed to search all three ports. When a dog searched the three ports and indicated on the food port, the clicker was activated, and the dog received a food reward. Once a dog was able to successfully find, and indicate on, the food port for a minimum of eight of out 10 trials, the food port was phased out. Here, the food item was removed from the port, but the port was not cleaned so that the breath and sweat sample was paired with a residual food odour. When a dog could successfully indicate on this port in a further eight out of 10 trials, a novel clean port that had never held food was then introduced. Here, the dog was presented with three clean ports: one held a breath and sweat sample whereas the other two held blank samples. The dog was rewarded for correctly alerting on the port that held the breath and sweat sample. This process was not always linear, and, if a dog was struggling to correctly identify the clean port containing the breath and sweat sample, the food port could be intermittently re-introduced during this initial stage of training to increase confidence and performance. Once the dog was successfully achieving a minimum of eight out of 10 trials on clean ports, the food ports were no longer used, and the dog progressed to discrimination training.

#### 2.5.2 Training: Between people sample discrimination

This study utilised a two-phase three-alternative forced choice procedure. The phases consist of Phase One: learning the target odour, and Phase Two: discrimination. In Phase One, ten trials were run in which the dog was shown their target sample (a breath and sweat sample collected by Person A), alongside two blanks (unused gauze). At each stage, the “blanks” were created (inserting a piece of unused gauze into a vial) by the participant providing the samples (whilst wearing nitrile free gloves) immediately prior to providing their breath/sweat sample to avoid odour contamination. Phase One showed the dog what their target odour was for that session, as it was the only human odour present in the line-up. The location of the target odour (port one, two or three) was generated in a pseudorandom order using a random number generator selecting between one and three. The location was pseudorandom because it followed constraints: the target could not be located in the same position for more than two trials consecutively and the target had to be in each of the locations at least three times across 10 trials. If a dog performed their alert behaviour on the target sample in at least seven out of 10 trials (binomial probability of this occurring by chance *p* < 0.05), they progressed to Phase Two.

In Phase Two, 20 trials were run in which the dog was shown their target sample (the sample that they had been rewarded for alerting on in the first 10 trials), a sweat and breath sample from a different person, Person B (the distractor), and a blank. Both people who provided samples had not undergone any stress inducing protocol when the samples were taken as we were merely asking the dog to continue to find the first odour presented to them, in this case Person A’s odour (target) instead of Person B’s odour (distractor). The dog was required to continue to indicate on the target sample (Person A’s odour), despite a second human breath/sweat odour (Person B’s odour) being present in the line-up. This method is conceptually similar to (although not methodologically identical to) a match-to-sample task, in that the dog learns what their target sample is for that session in the first ten trials, and, in the next 20 trials, a second odour is added into the line-up. During training, we used samples with known odour differences between the target and the distractor to establish each dog’s ability to perform the paradigm before moving onto undetermined samples at testing (baseline and stress). If the dog is successful in continuing to find the target odour during the discrimination trials it demonstrates that the target and distractor odours are distinguishable to the dog.

If the dog provided their alert behaviour on the target sample, the clicker was activated and the dog received a food reward. If the dog alerted on the distractor or blank, the handler did not respond until the correct choice was made. During training, the dog would be recorded as giving a false alert, but they were assisted in finding and, correctly alerting on, the target. The number of correct trials to be considered statistically above chance is 12 out of 20 (*p* < 0.05). However, as discriminating between two different people’s breath/sweat is an easier modification of the final task, we used a stricter criterion during this training stage. We required dogs to successfully indicate the target sample in 16 out of 20 trials (80% correct trials, *p* < 0.001), for two consecutive sessions, before progressing to the next training stage: within person sample discrimination. Each session, a dog was shown two novel individual’s samples, and the person chosen as the target was decided using a random number generator (where Person A and Person B are assigned numbers one or two, one = target, two = distractor). Samples were disposed of at the end of each session and were never re-used.

#### 2.5.3 Training: Within person sample discrimination

**Fig 4:**
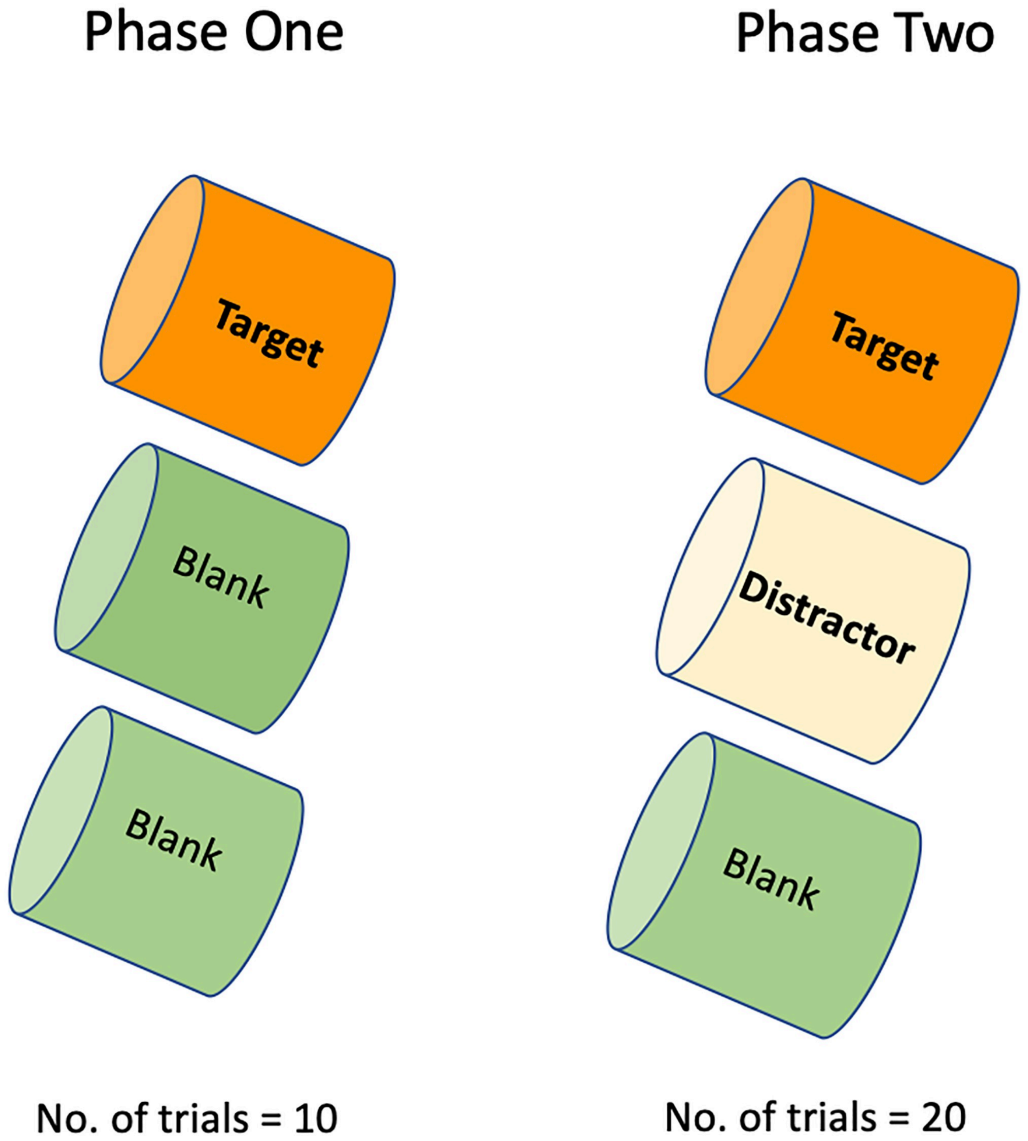
Diagram illustrating the general two phase, three alternative forced-choice procedure. Each cylinder represents a port as shown in Fig 1.

In this stage of training, dogs were presented with one person’s breath taken at two different times of day (e.g., first thing in the morning before brushing teeth and mid-afternoon) (see Fig 4). The sample collected in the morning was typically used as the target sample and was presented to the dogs following Phases One and Two described above, but here the distractor sample was the same person’s sample from the afternoon. In this instance, the background VOC profile of the samples came from the same person, however there were presumed distinctions in the exogenous VOCs due to time passing, the person having consumed food and drink, and so on, between sample collections. Criteria for success in this stage of training was the same as described in Section 2.5.2 for training between people sample discrimination. During training within-subject discrimination, the double-blind procedure was also introduced. The researcher who knew the location of the target sample moved to be out-of-sight to the dog (for further details see 2.7.2). If dogs were successful (7/10 on Phase One and 16/20 at Phase Two) for a minimum of two sessions (e.g., they could successfully discriminate within-subject for a minimum of two different people) they could proceed to testing.

### 2.6 Testing: Within person baseline and stress sample discrimination

**Fig 5:**
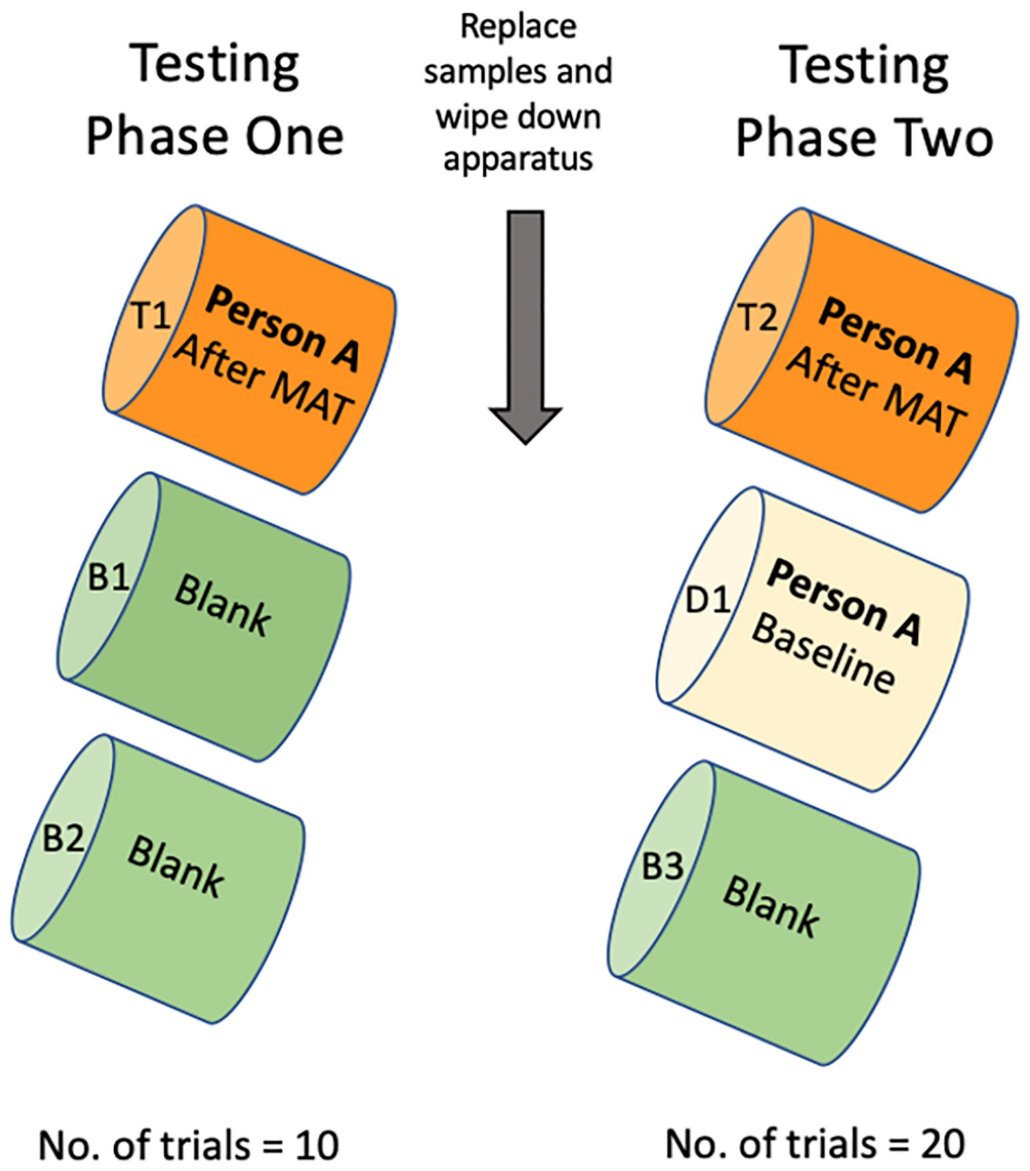
Diagram illustrating the within-subject test design, where the target = stress sample (Person A immediately after MAT), distractor = baseline sample (Person A, four minutes before, at baseline), blank = unused gauze in a glass vial.

During testing, dogs were presented with each participant’s samples using the two-phase procedure outlined previously. At this stage, the target sample was the combined breath and sweat taken immediately after a participant completed the MAT and the distractor sample was the combined breath and sweat taken from the same participant at baseline, prior to taking part in the MAT (Fig 5). As before, each session consisted of 10 training trials in Phase One (target, blank, blank) and 20 discrimination trials in Phase Two (target, distractor, blank). For all training and testing, samples were taken within three hours of being shown to the dog and were stored at room temperature. Each participant’s samples were used in a single session (shown to one dog only) and then disposed of. During this stage, if a dog made an incorrect indication, they were recorded as giving a false alert on that trial, called away from the apparatus, and the next trial was prepared. As such, dogs were never assisted into making a correct alert and the handler was blind to the location of the target.

### 2.7 Controls

#### 2.7.1 Odour

During sample collection, and for all handling of the ports, nitrile powder free gloves (Unicare non-sterile, single use examination gloves) were worn. During discrimination training and testing, the dogs were presented with fresh samples at the start of the Phase Two (discrimination trials). To do this, multiple samples were taken at the same time point. During participant testing, three blanks were made (B1, B2, B3), one distractor at baseline was taken (D1), and two targets at the end of the MAT were taken (T1, T2). The order that these were presented to the dog were: Phase One: T1, B1, B2, Phase Two: T2, D1, B3 (Fig 5). In doing so, when the discrimination trials started, all three samples were fresh, which eliminated the possibility that a dog could follow their own scent from Phase One or rely on odour differences associated with when the sample had been opened. A dog following their own odour can be described as them ‘tagging’ the port, meaning that they are aiding their discrimination by adding additional cues outside of the experimental design (e.g., licking the target port). To further reduce the likelihood of tagging, ports were wiped down with isopropanol (diluted with distilled water to 70% concentration) between each trial. In both training and testing, no samples were re-used or shown to more than one dog. This safeguards from potential issues of odour degradation or dogs incorporating the scent profile of the other study dogs into their decision making. These controls relate to potential extraneous cues associated with the samples themselves. A further area of concern is the possibility of extraneous cues coming from the apparatus itself, for example, the port that is designated to hold the target accumulates a distinctive odour over time, or there is a visual difference specific to that port that the researchers are unaware of. To control for this, all arms and ports of the apparatus were replaced with a new set once during the study. Because the fourth dog reached testing after the apparatus was replaced, one control session was conducted in which target and distractor arms were switched: the arm/port that previously held the target sample (the dog would get rewarded for indicating on this) was switched to holding the distractor sample (the dog would not get rewarded for indicating on this and it would be considered a false alert) and vice versa. If dogs were relying on extraneous cues provided by the apparatus, we would expect to see a decrease in performance after implementing the new apparatus (for three dogs) or switching which arm held the target sample (for one dog). Possible impacts on the dogs’ performance due to these controls were analysed.

#### 2.7.2 Visual

During within person training, and throughout testing, a double-blind procedure was used. Here, the first researcher (hereby referred to as the handler) was visible to the dog but the handler did not know where the target odour was located. The dog indicated their choice and the handler relayed this information to the second researcher who was out-of-sight (behind a 260x180cm three-panel room divider). The out-of-sight researcher confirmed this alert as correct or incorrect and the handler activated the clicker and rewarded the dog, or ended the trial, depending on the researcher’s response. This system prevented the handler from providing any unconscious cues to the dogs, and decreased the likelihood that the dogs would pick up on extraneous visual cues that may influence their performance.

### 2.8 Statistical analyses

#### 2.8.1 Human self-report scores

The participants’ VAS scores were downloaded from Qualtrics and inputted to Microsoft Excel version 16.44. Each participant’s pre-task VAS stress score was subtracted from their post-task VAS stress score to establish whether their score had increased at least two points as a result of the MAT.

#### 2.8.2 Human physiological data

Physiological data were scored using AcqKnowledge software (Biopac Systems, Goleta, CA). Heart rate and mean arterial pressure (i.e., blood pressure) were calculated by taking the mean of the last two minutes of the baseline period and the mean of all three minutes of the MAT period. The first minute of the baseline period was excluded from the analysis on the basis that there may be spurious readings as a result of the participant recently standing up and moving while the sensors were put on, and small movements in the first minute of sitting down while they became accustomed to the sensors. Physiological reactivity was determined by subtracting averaged baseline activity from the MAT period activity. Whether the participant met both the self-report and physiological criteria to use their samples in testing was assessed using these methods immediately after completing each participant’s session, as this information was required to determine whether to show the samples to the dog, or whether they should be disposed of.

#### 2.8.3 Dog performance

Dogs’ performances were analysed using binomial probabilities to determine if they could detect the target odour at levels above chance. The probability of choosing the target odour by chance on any given trial was 0.33. The alpha level was set at 0.05. To examine the extent that dogs may be learning within the discrimination trials, the first discrimination trial only was additionally assessed (each dog’s first exposure to each participant’s T2, D1, and B3 samples).

#### 2.8.4 Impact of odour controls

To assess for the possibility that dogs were using extraneous cues specific to the apparatus to aid in their indication decisions, we analysed the dogs’ performances on the sessions prior to- and post- the odour control intervention (replacing the arms for three dogs and switching the ‘correct’ arm for one dog). Trial scores and mean performance are reported. A repeated measures t-test was run on the number of correct trials on each dog’s last session using the original apparatus and their first session using the odour control. SPSS version 28 was used for this analysis with the alpha level set at 0.05.

## 3 Results

### 3.1 Human samples

Fifty-three participant’s samples were collected: 13 remotely and 40 in-person. Of those, 11 were excluded because the participant did not meet the criteria of experiencing negative stress in response to the MAT (two remote participants and nine in-person participants). The basis of exclusion due to the stress criteria were as follows: five reported no, or less than a two-point, increase in their self-report experience of stress following the MAT, five showed a decrease in BP following the MAT (indicative of a positive stress response, or “challenge” [15]), and one showed a decrease in HR following the MAT (see S1 Dataset for full list of samples taken and details on their exclusion). A further five participants’ samples were excluded: three because they were surplus to the number of dogs able to be shown samples to that day, one because the participant did not meet our criteria regarding food (had chewing gum in their mouth), and one because the participant withdrew during the task. One test session ended after ten trials because the dog was unwell at testing. In total, 36 participants’ samples were tested.

The 36 tested samples were collected from 30 females and six males with a mean age of 25.42 years (Min = 18, Max = 57). Participants included 30 people who identified as White, three as Asian or Asian British, two as Mixed or multiple ethnic groups, and one as Black, African, Caribbean or Black British. In the baseline condition, the mean self-report VAS stress score (minimum possible score: 0, maximum possible score: 10) was 1.98 (Min = 0.00, Max = 6.51). The mean self-report VAS stress score immediately after the MAT was 7.00 (Min = 3.00, Max = 10.00). For the 25 in-person samples for which physiological data were additionally recorded, the mean heart rate during baseline was 90.54bpm (Min = 66.61bpm, Max = 115.58bpm), and during the MAT was 104.91bpm (Min = 81.30bpm, Max = 130.50bpm). Mean blood pressure (i.e., mean arterial pressure) during the baseline period was 114.50mmHg (Min = 92.66mmHg, Max = 164.95mmHg) and during the MAT was 123.21mmHg (Min = 92.81mmHg, Max = 172.34mmHg) (see S1 Dataset for details of HR and BP per participant).

### 3.2 Dog performance

Per test session, each dog completed 10 Phase One trials (T1, B1, B2) and 20 Phase Two discrimination trials (T2, D1, B3). The focus of the reported results will be the 20 discrimination trials where the dogs were discriminating between each participant’s baseline and stress samples (for results of the Phase One trials see S1 Dataset). Due to the availability of collected participant samples coinciding with the dog owners’ schedules, each dog participated in a different number of test sessions. Each session indicates a single participant’s samples (e.g., 36 sessions = 36 within-subject tests). The dogs’ overall performances can be seen in Table 1. Each dog’s performance in individual sessions can be seen in the S1 Dataset. As a cohort (N = 36 sessions, 720 discrimination trials), the dogs alerted to the stress sample in 93.75% of trials. A binomial test, where the probability of success on a single trial is 0.33, found each individual dog’s proportion of correct trials was greater than that expected by chance (*p* < 0.001), and the combined cohort proportion of correct trials (675/720) was greater than that expected by chance (*p* < 0.001) (Table 1). When looking at each dog’s first discrimination trial performance only, Treo scored 100% (16/16 correct identification of the stress sample), Winnie scored 100% (2/2), Fingal scored 90.91% (10/11, one false alert on a participant’s baseline sample) and Soot scored 85.71% (6/7, one false alert on a participant’s baseline sample). Overall, dogs correctly alerted on the stress sample in 94.44% (34/36) of first discrimination trials.

**Table 1:** Each dog’s performance discriminating between baseline and stress samples NB.

Dog name	Number of test sessions	Total number of discrimination trials	Performance range	Average performance across all test sessions	Mean correct trials (SD)	95% Confidence Interval (lower, upper)	*p* value (Binomial test)
Treo	16	320	70–100%	96.88%	19.38 (1.54)	18.62, 20.13	*p* < 0.001
Soot	7	140	75–100%	92.14%	18.36 (1.29)	17.60, 19.13	*p* < 0.001
Fingal	11	220	85–100%	90.91%	18.42 (1.81)	17.09, 19.77	*p* < 0.001
Winnie	2	40	85–95%	90.00%	18.00 (1.41)	16.05, 19.95	*p* < 0.001
Total	36	720	70–100%	93.75%	18.81 (1.55)	18.30, 19.32	*p* < 0.001

Number of discrimination trials (max possible correct score) per session = 20.

### 3.3 Odour controls

The dogs’ performances were compared pre- and post-odour controls to assess for the potential impact of confounding odour cues informing their performance. The original apparatus and arms had been used for all training sessions prior to testing (with the exception of the “food port” arms which had been excluded and stored away once the dog had progressed to “clean” ports). Treo, Fingal, and Soot had the arms replaced after twelve, two and one testing sessions, respectively. As Winnie joined the study after the apparatus had been replaced, her session scores pre- and post-switching which arm held the target sample are reported. Each dog’s performance pre- and post-odour control can be seen in Table 2. As a cohort, results of a repeated-measures t-test showed a non-significant difference in the number of correct trials in the last session pre-odour control (*M* = 18.25, *SD* = 1.50) and the first session post-odour control (*M* = 19.00, *SD* = 0.82), t(3) = -1.00, *p* = 0.391. These results indicate that the dogs were not relying on additional odour cues to inform their decisions.

**Table 2:** Odour control data for each dog.

Dog name	Original apparatus test session n	Original apparatus mean performance (SD)	95% CIs	New apparatus test session n	New apparatus mean performance (SD)	95% CIs (lower, upper)	Last session original apparatus score	First session new apparatus score
Treo	12	19.25(1.76)	18.25, 20.25	4	19.75(0.50)	19.26, 20.24	20	20
Fingal	2	18.00(1.41)	19.26, 20.24	9	18.44(1.33)	17.57, 19.31	19	18
Soot	1	17.00(NA)	NA	6	18.67(1.86)	17.18, 20.16	17	19
							First Session Score	Switched D and T Arm Score
Winnie	-	-	-	-	-	-	17	19

## 4 Discussion

This is the first study to use a controlled olfactory paradigm to assess if dogs can discriminate between human odours (combined breath and sweat samples) taken at baseline and when experiencing experimentally induced negative psychological stress. To test this, we trained dogs on a two-phase, three-alternative forced-choice paradigm of increasing difficulty, initially on odour discriminations with known VOC differences: between people discrimination, progressing to within person discrimination (the same person at two times of day). Performance at above chance level (80% correct, *p* < 0.001) was required at these training stages before reaching testing. By doing this step-wise method, we could assume that if a dog’s performance dropped to chance at the testing stage, it was because the stress and baseline samples were indistinguishable to the dog, and not because the dog did not know how to complete the task. The results on the test samples showed that, in tests of discrimination, the dogs’ performances were consistently above chance, ranging between 90% to 96.88%, with a combined performance of 93.75% correct trials across sessions.

To analyse the significance of the dogs’ responses using paradigms such as this, multiple trials are required to gain statistical power (the probability of a dog guessing correctly on a single trial is one in three). As such, each dog carried out twenty discrimination trials within each session to assess their ability to discriminate between the samples. However, of additional interest is each dog’s first exposure to the three, newly opened, samples at the beginning of the discrimination phase (T2, D1 and B3). Analysing this trial in isolation provides information on whether the dogs could discriminate between an individual’s baseline, stress and blank sample by recognising that the newly opened stress sample (T2) is the same odour profile that was reinforced in the learning trials (T1), while concurrently recognising that the baseline sample (D1) is distinct from what they have previously been rewarded for and should be passed over. We found that the dogs were highly successful in the first trial of each session’s discrimination phase, and correctly alerted on the stress sample in 94.44% of first exposure trials. Indeed, the dogs incorrectly alerted on the baseline sample in their first exposure on only two occasions. Even if the dogs had shown high rates of false alerts in their first exposure but had gone on to learn to discriminate throughout the twenty trials, this would still indicate that the two odours are able to be distinguished, however, this could raise questions into whether the dogs were developing extraneous cues (e.g., adding their own odour to the sample) to aid in their discrimination across the repeated trials. However, our finding that the dogs were able to discriminate on first exposure provides strong evidence that the samples conferred distinctive odour profiles. Overall, the dogs’ performances indicate that each participant’s samples were distinct at baseline compared to after the stress induction. Furthermore, the results of the odour control procedures (replacing the apparatus and switching the target arm) suggest that the dogs were not relying on extraneous or confounding cues to aid in their discrimination of the samples. These results corroborate the findings of other studies (e.g., [7, 28]) suggesting that dogs are able to detect human physiological changes associated with psychological states and, further, highlight the importance of considering transmission of odour cues in both pet dog ownership and service dog training.

The current study adds to the field by controlling for potential odour confounds that have not been explicitly controlled in previous studies. For example, D’Aniello et al. [7] found that dogs showed more stress-indicative behaviours when in a room with a stranger and exposed to odours samples from people experiencing “fear” compared to when the dogs were in the same room with a stranger and exposed to odours of people when they were “happy”. There are two areas to consider; first, no physiological measures were used to confirm the emotional states of people donating the biological samples, and second, the samples that represented the different emotional conditions were donated one week apart, thus increasing the likelihood of confounding odours (e.g., due to diet, medication, changes etc.), regardless of the emotional states they were meant to represent. The current study addressed both of these areas by corroborating the participant’s self-reported stress with physiological measures (for 25 of 36 samples) and imposing strict odour controls to minimise the potential impact of extraneous variables assisting in the dog’s discrimination. For example, samples were taken from each participant in the same room within four minutes of each other, reducing the likelihood that dogs were able to inform their indication decisions by using background VOCs from the air in the room or exogenous VOC changes in the participant due to time passing. It should be noted, however, that the focus of D’Aniello et al.’s [7] study was emotional contagion, which was not our focus, as we trained dogs explicitly for discrimination purposes. D’Aniello et al. [7] reported more stress-related behaviours exhibited by dogs in the condition where they were presented with human sweat samples taken when they reported to be experiencing “fear”, suggesting that, not only could detect an odour, but it also had a mirroring effect on the dog’s own emotional state. It is possible that dogs in the current study were able to recognise the odour of stress as having an emotional context, however the positive reinforcement training likely interfered with this interaction. Although dog behaviour was not coded in this study, it can be anecdotally noted that no dog showed signs of distress when encountering the human stress samples. On the contrary, dogs appeared excited when they came to the stress sample as they were anticipating the clicker and food reward for a correct alert. Future studies may wish to examine the interaction between the emotional contagion of stress and positive reinforcement directly to add insight into this area. The current study does, however, provide evidence that dogs can detect an odour associated with acute stress in humans from breath and sweat alone, which provides a strong foundation for future investigations into areas such as emotional contagion knowing that there is a confirmed odour component to acute negative stress that can be detected in the absence of other visual or vocal cues.

The results of this study add to previous research identifying VOC changes in humans who are experiencing acute stress (e.g., [52–54, 57]) by further demonstrating that dogs can detect this VOC profile change. As posited by Turner et al. [53], there are likely VOC changes associated with increased breathing rate, heart rate and blood pressure. These responses are, in part, due to a cascade of hormonal release associated with stress. The most well-known is the secretion of cortisol, but there is also the stimulation of gluconeogenesis, glycogenolysis, and lipolysis, and increased levels of renin and angiotensin II enzyme [53, 63]. Many of the physiological markers associated with a stress response are influenced by factors other than stress, for example, physical exercise, tobacco, alcohol, and the time of day [64, 65]. Because of this, a within-subject design utilising exclusion criteria minimises the potential for these physiological markers being impacted by anything other than the stress-inducing task.

We did not attempt a definition of stress from set values of HR and BP across individuals (e.g., “HR must raise to above 100 beats per minute”) because each individual’s baseline HR and BP is contributed to by many factors and imposing such measures would be largely arbitrary. However, we did see notable differences in the actual values of baseline and stress condition HR and BP between participants. It is possible that some participants were emitting bio-markers of stress during the baseline condition in addition to after the MAT. However, given that we required a two-point increase on their self-report stress scale and an increase in HR and BP, this meant that we excluded both participants who were “not stressed” at baseline and after the MAT, and also those who were “stressed” at baseline and after the MAT, as neither group would have shown change between conditions. The varying baseline values of HR and BP do, however, raise interesting questions on what specific biomarkers the dogs may be detecting. Future studies combining VOC analysis using GC-MS and dog detection using samples taken at the same time point from the same participants would be beneficial. It should be noted that in Santos et al.’s [54] study assessing VOCs in participant’s breath after a stress-induction task, additional breath samples were taken at two time points after the task had ended: five minutes, and one hour, afterwards. The observations related to the samples obtained at these time points showed no distinguishable VOC pattern between relaxed and stressed conditions. This finding suggests that the VOC pattern observed was associated with metabolic pathways of the acute stress response, which ceased to become detectable once this process was no longer taking place. Cortisol is the most abundant glucocorticoid released in response to a stressor, and is generally acknowledged as the ‘stress hormone’. However, cortisol levels peak approximately 10 to 30 minutes after an acute stressor [66], raising questions into what metabolic processes the dogs are actually detecting. It is likely, as our samples were taken immediately upon finishing the stress inducing task, that dogs were utilising VOC changes associated with the acute stress response, however, further investigations into dogs’ performances using samples taken five minutes to one hour after a stress inducing task had ended would add insight into the timeline of the physiological response. Establishing at what point dogs can no longer distinguish between samples may add to our understanding of the scent profile of stress.

It must be noted that our sample consists of only four dogs. This sample size is, however, in-line with other bio-detection studies due to the time-consuming nature of training highly specialised dogs (e.g., Cornu et al. [41]: one dog; Bomers et al. [43]: one dog; Taverna et al. [42]: two dogs; Murakra et al. [40]: four dogs, Kantele et al. [45]: four dogs). Importantly, as this is a proof of principle study, a small sample size does not compromise the findings, as the goal of the study is not to generalise the findings to all dogs, but rather to demonstrate that some, carefully selected and highly trained dogs can successfully discriminate between the samples. To provide evidence that a small number of dogs can detect odour differences in baseline and stress samples suggests that an odour difference exists.

Moreover, while the two-phase within-subject paradigm utilised here is not widely used in the existing bio-detection literature, it provides well-controlled evidence that two odours are distinguishable from each other while minimising the need for a large number of human samples required to teach a generalised odour concept. With this in mind, the two-phase within-subject protocol could be recommended for future studies investigating physiological changes within an individual if access to a large number of samples is not feasible, or the ability to store samples for later use is limited. In terms of participant samples, a total of 36 is strengthened by the within-subject nature of the design. Each participant acted as their own control, minimising potential issues of variance from background VOCs associated with age, sex, ethnicity, diet and lifestyle.

When considering the stress-inducing task itself, we should highlight that it failed to induce negative reactions to stress in 11 participants (21%). This may be due to the participants’ perceptions of the stress-inducing task. The biopsychosocial model of challenge and threat posits that stress may lead to either positive or negative outcomes depending on how an individual perceives the stressor [14–16]. Negative responses to stress (threat) were observed in the majority of our sample, and occur when an individual does not believe they have the resources to overcome a stressor and is accompanied by increased heart rate and blood pressure. However, of the 21% who did not meet our criteria for stress, five did not interpret the task as stressful (i.e., did not report an increase in VAS score of at least two points), and a further five appeared to have experienced positive stress (challenge), as they showed an increase in VAS score and HR, but a decrease in BP [15]. If future studies wished to replicate, or extend, our findings we recommend examining how these different stress responses may impact detection of VOCs, or explore manipulations to induce negative stress more consistently (e.g., Trier Social Stress Test [67]), saving researchers time and expense on unusable samples.

While the potential issue of confounds due to unintentional visual cues have been highlighted and addressed in many canine bio-detection studies (e.g., [24, 41]), and discussed in review papers [68], it is less common to see implementation of additional odour controls. It is possible that, over time, the dog learns to integrate extraneous cues, relating to either the samples or the apparatus. Lazarowski et al.’s [69] methodological review highlights this issue (although broadly focussed on odour generalisation paradigms), and we reiterate the importance of attention brought to potential extraneous cues, beyond visual cues, in olfactory paradigms. We believe that it is important for future papers to include information specific to the reduction of extraneous odour cues, and to implement odour controls as consistently as visual controls.

The results of this study contribute to our understanding of human-dog relationships and add insight into how dogs may be interpreting their environment and interactions with humans as informed by their olfactory capabilities. Our findings demonstrate that there is a detectable odour associated with acute negative stress that is distinct from odours at baseline. While the dogs in this study underwent training in order to communicate that they were able to distinguish between odours, the found performances on this task suggests that there are VOC changes induced by acute negative stress that are detectable by dogs. Having established that there is an odour difference, assessing how untrained dogs may recognise and interpret these odours could be of interest in future studies. The results of the current study could have further applications to the training of anxiety and PTSD service dogs, that are, currently, predominantly trained to respond to visual cues [70]. Knowing that there is a detectable odour component to stress may raise discussion into the value of olfactory-based training (e.g., taking samples from a person when relaxed and experiencing stress) and positively reinforcing the dog to attend, or perform attention seeking behaviours in response to, this odour (similarly to how Medical Assistance Dogs are trained). As the current study was laboratory based, such methods would need to be tested in applied situations for this premise to be verified. It is important to consider the welfare of service dogs tasked with performing these types of roles, especially in light of potential emotional contagion between owner and dog. A recent study by van Houtert et al. [71] tested whether service dogs supporting in stress reduction for veterans showed higher levels of hair cortisol (a proxy measure of stress) as compared to companion dogs. They found that cortisol values did not differ between service and companion animals, which may seem at odds with the results of Sundman et al.’s [28] study which found that owner and dog cortisol levels mirrored each other. Crucially, service dogs receive specific positive reinforcement counter-conditioning training that is designed to counteract any untrained stress contagion. Indeed, van Houtert et al.’s [72] study found that service dogs’ cortisol levels reduced after a training session. More research into the interaction between stress contagion and positive reinforcement training is needed, however van Houtert et al.’s [71, 72] findings suggest that, with appropriate training, the issue of emotional contagion on trained service dogs may be counteracted. Service dogs for those with anxiety, panic attack disorders and PTSD are growing in popularity and the results of this study confirm that trained dogs are able to detect the physiological processes associated with an aspect of these conditions from odour alone.

In conclusion, dogs were able to discriminate, with a high degree of accuracy, between human breath and sweat samples taken at baseline and when experiencing psychological stress. These results suggest that there is a VOC profile associated with acute psychological stress that is detectable by trained dogs. Having established that this odour is detectable, further investigations may wish to apply this to real-world settings. It is possible that an odour component may be useful as a training aid for service dogs tasked with responding to acute stress responses in their owner. More broadly, establishing that dogs can detect an odour associated with stress sheds light on the human-dog relationship and adds to our understanding of how dogs may interpret, and interact with, human psychological states. Further studies are required to establish what exact odour the dogs are detecting, the time-frame that this odour is detectable for, and potential interactions with chronic or long-term stress responses.

## Supporting information

S1 Dataset Full participant sample dataset with dog performance scores. (XLSX)
